# HARMONIZATION OF MEDICARE BENEFICIARIES’ COGNITIVE FUNCTION DATA

**DOI:** 10.1093/geroni/igae098.2181

**Published:** 2024-12-31

**Authors:** Anum Zafar, Fred Kobylarz, Rebecca Zaritsky, Hyosin Kim, Maria Lopez, Weiyi Xia, Haiqun Lin, Olga Jarrín

**Affiliations:** Rutgers, The State University of New Jersey, New Brunswick, New Jersey, United States; Rutgers Robert Wood Johnson Medical School, New Brunswick, New Jersey, United States; Rutgers, The State University of New Jersey, New Brunswick, New Jersey, United States; Oregon State University, Corvallis, Oregon, United States; Rutgers, The State University of New Jersey, New Brunswick, New Jersey, United States; Rutgers, The State University of New Jersey, Piscataway, New Jersey, United States; Rutgers, The State University of New Jersey, Newark, New Jersey, United States; Rutgers, The State University of New Jersey, New Brunswick, New Jersey, United States

## Abstract

This study explains the methodology to harmonize data from healthcare claims and clinical assessments across various post-acute and long-term care settings for Medicare beneficiaries who died in 2019, age 50 or older at death, with at least five years of continuous Medicare enrollment. The cognitive function trajectory file was constructed from 2014-2019 Medicare and Medicaid claims and encounter files (ICD-9 and ICD-10 codes), and cognitive assessments from post-acute and long-term care settings (BIMS scores and other relevant items from MDS, MDS-SB, OASIS, and IRF-PAI assessments). Cognitive function data were harmonized to create a four-category variable: no impairment, memory loss, mild cognitive impairment (MCI), and dementia/severe cognitive impairment. Data were summarized for each quarter (3-month period) of the last five years of life (20 timepoints). Of the nearly two million beneficiaries in our study population, 19.4% had no evidence of cognitive impairment at any point. Five years before death, 3.2% of the sample population had mild cognitive impairment (MCI) and 7.7% had dementia or severe cognitive impairment. At the time of death, 14.8% of the sample had MCI and 50.6% had severe cognitive impairment/dementia. Among beneficiaries with any cognitive impairment, claims or assessment data contributing new information to the trajectory file were available for an average of 7 (out of 20), s.d. 5.7 quarters, median=5, inter-quartile range: 2-11. The harmonized longitudinal dataset allows for tracking change in cognitive function and identifying risk factors for cognitive decline.

